# Non-aneurysmal subarachnoid haemorrhage in COVID-19

**DOI:** 10.1007/s00234-020-02535-4

**Published:** 2020-08-28

**Authors:** Suzanne Harrogate, Alex Mortimer, Lorna Burrows, Barnaby Fiddes, Ian Thomas, Claire M Rice

**Affiliations:** 1 North Bristol NHS Trust, Southmead Hospital, Bristol, UK; 2 Translational Health Sciences, Bristol Medical School, University of Bristol, Bristol, UK

**Keywords:** Subarachnoid haemorrhage, COVID-19, Clinical neurology, Neuroradiology

## Abstract

Coronavirus disease of 2019 (COVID-19) is associated with hypercoagulopathy, but haemorrhage, including spontaneous intracerebral parenchymal haemorrhage and diffuse petechial cerebral haemorrhage, has also been reported. We present two cases of nonaneurysmal subarachnoid haemorrhage (SAH) in patients with severe COVID-19. Careful review of neuroimaging for haemorrhagic complications of COVID-19 should be undertaken, particularly for those patients receiving enhanced prophylaxis for venous thromboembolism. Although likely to be a marker of severe disease, non-aneurysmal SAH can be associated with favourable outcome.

## Introduction

Although a predisposition to thrombosis in COVID-19 is well-recognised [1, 2], reports of haemorrhage affecting multiple organ systems, including the CNS are emerging [3, 4]. The pathophysiology is not yet fully understood and may be multifactorial including microthrombosis with secondary haemorrhage, dysregulated coagulation, e.g. disseminated intravascular coagulation and immune thrombocytopenic purpura [5], and vascular hyperpermeability in the context of COVID-19 cytokine storm [1], endotheliitis [6] and vasculitis [7].

We report 2 cases of non-aneurysmal SAH in severe COVID-19 and hypothesise that this occurred as a complication of thromboembolic disease in the context of enhanced prophylaxis for thromboembolic disease. These cases add to the clinical spectrum of neurological complications associated with COVID-19 and highlight the importance of careful review of neuroimaging in patients with severe COVID-19.

## Patient A

**Fig. 1:**
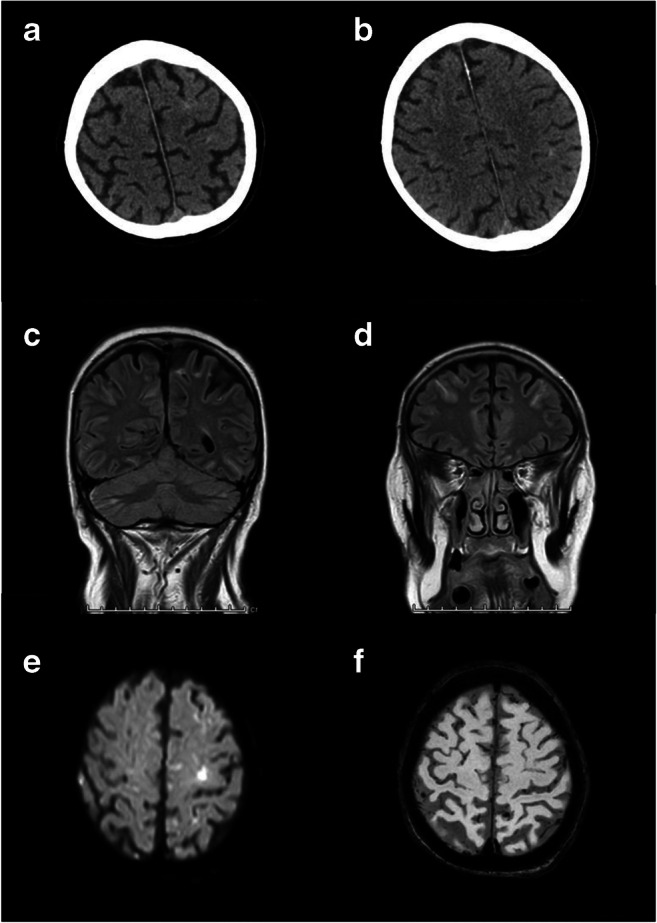
Non-aneurysmal SAH co-existent with COVID-19 (MRI). In patient A (**a**–**f**), brain MRI results demonstrated multifocal convexity high signal change consistent with acute subarachnoid haemorrhage and small infarcts including in the left precentral gyrus (**a** and **b** axial T1, **c** and **d** coronal fluid-attenuated inversion recovery, **e** axial diffusion, **f** axial susceptibility weighted imaging)

**Fig. 2:**
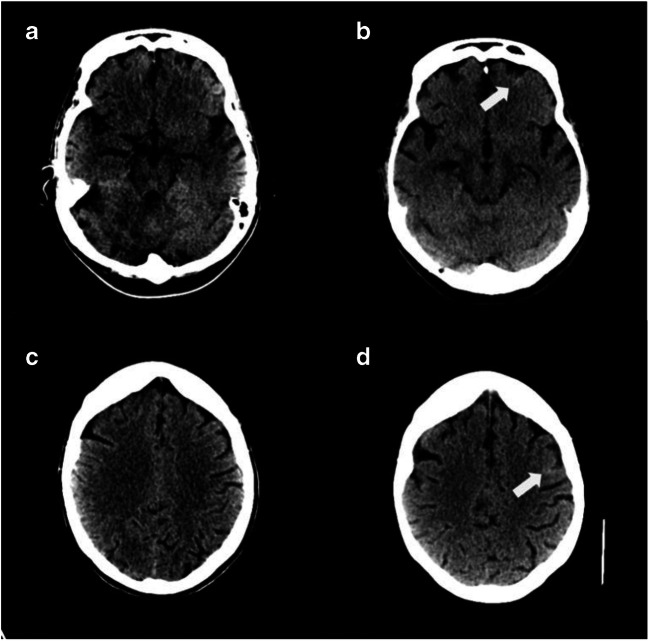
Non-aneurysmal SAH co-existent with COVID-19 (non-contrast CT). Contemporary non-contrast CT brain imaging for patient B was compared with that acquired 8 years previously and demonstrated multifocal, curvilinear foci of sulcal high density consistent with SAH (non-contrast CT head from 2011 **a** and **c**, 2020 **b** and **d** with arrows highlighting areas of high density)

A 74-year-old gentleman with confirmed COVID-19 and type 1 respiratory failure was admitted to the intensive care unit for mechanical ventilatory support. Maximum blood pressure was 130/55 mmHg. He received enoxaparin 40 mg bd subcutaneously as enhanced prophylaxis for venous thromboembolism [8]. Markers of disease severity included fibrinogen > 6 g/L, d-dimer assay value 55 μg/ml, ferritin 1808 μg/L and neutrophil:lymphocyte ratio 13. [9] At day 11, a CT head scan performed for reduced conscious level following sedation hold demonstrated multifocal, small volume, convexity SAH (Figs. 1 and 2). CSF analysis confirmed SAH (red blood cells 7614/mm^3^, white blood cells < 5/mm^3^, protein 1.1 g/L, negative for viral studies including SAR-CoV-2). The platelet count transiently fell 3 days prior to CT head scan (36 × 10^9^/L), but clotting studies were otherwise normal. A weakly positive lupus anticoagulant was detected together with anti-cardiolipin IgG antibodies (55 GPLU). Beta-2-glycoprotein 1 IgG and IgM were normal (< 10 U/ml). Paroxysmal atrial fibrillation was noted. Electroencephalogram was consistent with encephalopathy. An MRI brain scan demonstrated SAH with small multifocal infarcts and normal magnetic resonance angiography appearances (Fig. 1); SAH colocalised with areas of infarction but was also more widely distributed. On transthoracic echocardiography, a pedunculated mobile mass consistent with thrombus was seen within the left atrium attached to the inferior limbic band region. Blood cultures were sterile. Anticoagulation was held for 2 weeks, and interval neuroimaging excluded additional intracranial haemorrhage or new infarction following which he was fully anticoagulated. The mobile structure within the left atrium was reduced in size on follow-up transthoracic echocardiography. With continued supportive therapies, his level of consciousness improved, and he has been discharged for ongoing rehabilitation.

## Patient B

A 53-year-old gentleman with confirmed COVID-19 and type 1 respiratory failure required mechanical ventilatory support. Markers of disease severity included ferritin 2107 μg/L, fibrinogen > 6 g/L, d-dimer assay value 18.5 μg/ml and neutrophil:lymphocyte ratio 32. Maximum blood pressure was 152/60 mmHg. Enoxaparin was given 40 mg bd subcutaneously [8]. Raised clozapine level (4.6 mg/L, normal range 0.35–0.6 mg/L) was noted. CT head scan following seizure activity on sedation withdrawal demonstrated multifocal, curvilinear foci of sulcal high density consistent with SAH which was not present on historical imaging (Fig. 1). Coagulation studies were normal throughout. Electroencephalogram was consistent with encephalopathy. CSF analysis and MRI brain scan (complicated by motion artefact), performed > 7 days later, were normal. Blood tests were satisfactory with the exception of oligoclonal pattern on serum immunofixation. With continued supportive therapies, his level of consciousness improved. He has subsequently been discharged from the intensive care unit for continued rehabilitation.

## Discussion

We report 2 cases of nonaneurysmal SAH which have occurred in patients with severe COVID-19. We have attributed this to thromboembolism in the context of enhanced venous thromboembolism prophylaxis; alternative causes for non-aneurysmal SAH including reversible cerebral vasoconstriction syndrome, posterior reversible encephalopathy syndrome or cerebral amyloid angiopathy were considered unlikely based on the investigation results.

These cases highlight that, although prophylaxis and treatment of thrombosis associated with COVID-19 are undoubtedly important, clinicians should be alert to the possibility of haemorrhagic complications, particularly those associated with thromboembolic disease. Careful review of neuroimaging for subtle changes consistent with nonaneurysmal subarachnoid haemorrhage is required. Although likely to be a marker of severe disease due to COVID-19, SAH can be associated with a favourable outcome.
